# Environmental integration tool approach: Self-determined commitment and the adoption of pro-environmental behaviors

**DOI:** 10.3389/fpsyg.2022.903103

**Published:** 2022-09-23

**Authors:** Vincent Henin, Paula Uglione

**Affiliations:** ^1^Louvain Coopération, Université Catholique de Louvain, Louvain-la-Neuve, Belgium; ^2^Center for Development Studies, Institute for the Analysis of Change in Contemporany and Historical Societies, Université Catholique de Louvain, Louvain-la-Neuve, Belgium

**Keywords:** environmental integration tool approach, self-determined commitment, environmental behavioral intention, pro-environmental behavior, global south local economy actors, Belgian development cooperation, sustainable development

## Abstract

This paper discusses the role of self-determined commitment in the methodological dynamics of the Environmental Integration Tool Producer (EIT Producer) and its potential impact on the adoption of pro-environmental behaviors by local economy actors in the Global South. This tool is part of the EIT Approach developed by Louvain Coopération, the NGO associated with Université Catholique de Louvain (Belgium), which aims to support social actors in productivity and consumption activities in Africa, Andean America, and Asia. The aim is to highlight the conceptual and methodological elements of the OIE Producer, in order to understand the role of self-determined commitment in the expected ends and in the impact of the tool. The integration between economic development and the environment is a central issue in the challenges of contemporary global society. This article intends to contribute with answers to such challenges, especially with regard to the development of methodologies that are effective in governmental and non-governmental cooperation programs engaged for sustainable development.

## Introduction

In a global context, where the environment is at the heart of national and international agendas, governmental and non-governmental, the NGO associated with Université Catholique de Louvain (Belgium), Louvain Coopération, part of the Belgian development cooperation, began to build in 2011 a methodology for environmental integration called Environmental Integration Tool/ETI Approach (*Approche Outil d’intégration Environnemental* – *Approche OIE*). The EIT Approach offers a systematized transversality (although not mandatory) of the environmental theme in every phase of the Project Cycle Management. It consists of two tools – the EIT Program and the EIT Producer – created by Louvain Coopération to better meet the needs and objectives of its Food and Economic Security/FES Program projects by systematically taking into account their relationship with the environment. It is anchored in development principles according to which economic, social, and environmental aspects are inseparable and interdependent, and on research-intervention principles, that combines knowledge production, the transformation of social realities and the reinforcement of individual and social skills (Faure, 2010). The EIT-Program is implemented at the macro-level of a project/program and involves an analysis by the management team. The goal is to identify potential reciprocal relations between a project/program and its environment. The EIT Producer is designed to structure the dialogue between an economic agent who is responsible for a productive activity supported by a project/program (the Food and Economic Security Program, or others) and the management team of this program. However, it may also be employed by an unsupported economic agent, as an exercise of self-analysis. This economic agent may be an individual, a family, a group, an association, a cooperative, a local authority etc.

The EIT Approach was validated in 2019 from a participatory and multi-actor process (Henin and Uglione, 2021). The relevance and originality of the EIT Approach is recognized by different Belgium cooperation institutions like ARES (Académie de recherche et d’enseignement supérieur), VLIR-UOS, ACODEV (Fédération des Organisations de la société civile de Coopération au développement) but mostly DGD (Direction générale Coopération au Développement et Aide humanitaire). Built for the economic sector, it has also be adapted to Education sector by Educaid. Under the Food and Economic Security Program/FES, the application of the 1015 EIT Producer resulted in 1049 self-determined commitments by the local producers. Some examples of these commitments can be seen in initiatives in Benin, such as crop rotation, composting and usage of organic inputs and pesticides, integrated management of crop pests and diseases, reforestation, the use of living hedges and many others. In this country, this application generated 794 pro-environmental actions, which represents 77.31% of the commitments. The application of the EIT-Producer in Togo generated 89 pro-environmental actions by economic agents, which corresponds to 82% of the total self-determined commitments undertaken by these actors.

At this stage of development, a Document Analysis of the material of Louvain Coopération was proposed in 2020 with the objective of compiling the experience of creating these tools and assessing its impact, both for the consolidation of its practices and for the legitimization of the methodology disseminated. This research had as its historical boundary the period between the time of the launch of the EIT Approach in 2012–2020, when the mid-term evaluations of the 2017–2021 Food and Economic Security program were completed.

This paper presents the results of a qualitative research based on Documentary Analysis of materials on the EIT Approach with a focus on the methodological aspects of the EIT Producer variant. The aim is to discuss the role of self-determined commitment in the methodological procedures of the EIT Producer in order to identify the potential of the EIT Approach to influencing the adoption of pro-environmental behaviors by local economy actors. The purpose is to contribute to discussions about the methodologies of environmental integration in development cooperation programs and their impact on behavioral changes concerning the environment in the contexts where they are implemented.

## Framework

### Development cooperation

The origin of Belgian development cooperation is contemporary with the decolonization process after World War II, supported by the international instances of the United Nations. Since its beginning, Belgian development cooperation has been subject to criticism, several crises and political reforms (Contor, 2017). At the end of the 90s, a structural and political reform led to a major transformation of the sector. The Law on Belgian international cooperation (Kingdom of Belgium, 1999) clarifies the general objectives of Belgian cooperation such as sustainable human development and a longer-term political vision. Among its effects, the May 1999 reform confirmed the legitimacy of Belgian NGOs (actors of non-governmental cooperation) as representative structures of civil society and committed to the fight against poverty. The Administration générale de la Coopération au développement (now Direction générale Coopération au Développement et Aide humanitaire/DGD) presented a series of recommendations specific to the NGO sector. Among them, the strengthening of their social base, and the need to adopt a coherent approach and to work in complementarity with other actors. At the international level, NGOs are also becoming key actors in development cooperation (Charnovitz, 2002). NGOs are very important actors in Belgian cooperation, implementing development programs in the South or in Belgium (Molenaers et al., 2011). From 2013 onwards, environmental protection must permeate all interventions of the Belgian development cooperation (Law on Development Co-operation, Kingdom of Belgium, 2013). In 2014, the integration of environmental protection in the sectors of education, health, basic infrastructure and agriculture was elected as a priority for Belgian development cooperation (DGD, 2014). This priority is justified by the expectations of the Belgian development cooperation regarding the sustainable development of its programs and projects, understood as what ensures a just transition toward sustainable production and consumption patterns (Law on Development Co-operation, Kingdom of Belgium, 2013). The impact of environmental integration in development cooperation must be measured in terms of its contributions to sustainable development, and experiences have shown that this integration can provide important inputs to programs and projects, but that can also face many constraints (Ledant, 2016). Among them, a simplified or “minimalist” vision of sustainable development, which reduces its goals in relation to the current and future needs of societies. Also, the divergence between local and Northern environmental logics, particularly because the environmental impetus comes from donors and the integration orientations are based on Northern visions rather than on the rapports, desires and needs of Southern populations. This discrepancy gives environmental integration interventions an exogenous character, felt by the target populations in the South as intrusive and disturbing (Ledant, 2016). For the European Commission, the process of systematically integrating either a value, a theme or an idea into areas of cooperation requires changes in both ideas and practices. With regard to environmental mainstreaming, stakeholder participation is recognized as a key factor for sustainability in development cooperation (European Commission, 2011).

### Environmental behavior change

Environmental issues related to productivity and consumption patterns are an enormous and multidimensional challenge. Changing the behavior of social actors is part of this challenge. A number of factors can influence human actions; people contribute to their life circumstances, instead of being only passive products of them (Bandura, 2006). Environmental behavioral intention refers to the willingness of the individual to act and consume in a more sustainable manner. Environmentally friendly behavior is largely shaped by behavioral intentions (Braksiek et al., 2021). For Davis et al. (2009), commitment to the environment is a very strong predictor of pro-environmental behavior. According to the Transtheoretical Model/TTM of Prochaska and DiClemente (1992), adapted by Bamberg (2013), behavioral change is a gradual and non-linear process. Behavioral change intention is a crucial part of this process and depends on convincing arguments about the benefits of change (Richter and Hunecke, 2020). This process implies the passage of the individual through phases in which he/she has doubts regarding the advantages of change or his/her capacity to change. The intention to change has the function of a cognitive action plan by which the individual tries to eliminate or significantly reduce his doubts and ambivalence about the change and the new behavior to adopt (Prochaska and DiClemente, 1992). According to the Theory of Planned Behavior/TPB, by Ajzen (1991, 2011), individuals make reasoned decisions and this behavior is the result of the intention to engage in it. The intention to act is the direct antecedent of behavior (Ajzen, 1991). It constitutes the cognitive representation of a person’s willingness to adopt a certain behavior (Ajzen, 1991). Through intention, the individual foresees the consequences of his actions before deciding on taking them. Intention depends on three variables: attitudes toward the behavior, i.e., positive or negative perceptions of future behavior; subjective norm, or perceived social pressure; and perceived behavioral control, which provides a sense of how easy or difficult it is to perform the behavior (Ajzen, 1991). Other motivational factors such as personal norms and identity can also affect environmental intention behavior (Nigbur et al., 2010). The intention is an important predictor of people’s ecological behavior, and the stronger the intention, the more effort an individual will make to adopt pro-environmental behavior (Steg and Nordlund, 2013). Intention to act is a determinant of behavior toward the environment (Farrow et al., 2017). Regarding energy conservation there is a strong correlation between intention to act and real behavior (Macovei, 2015). The same is true for everyday behaviors such as turning off lights (Levine and Strube, 2012). A TPB-based study run in Saudi Arabia about the influencing factors on the intention to consume has found that environmental concerns, subjective norms, and attitudes were significant predictors of individuals’ adoption of hybrid electric vehicles (Alzahrani et al., 2019). These results are consistent with those of Lee et al. (2021) in Pakistan. The results of another study on the ecological behavior of biology professors showed that their knowledge of environmental issues indirectly influences their behavior through the intention to act (Iman et al., 2019). This is measured by the desire to protect the environment. Similar findings emerged from a study by Gkargkavouzi et al. (2019), which shows that knowledge could indirectly drive behavioral change by affecting the intention to engage in conservational efforts and could therefore be used to predict behavioral intention and environmental behavior. Research based on a cross-national social survey of 36 countries has shown that risk perception is a predictor of intention to act for the environment and that low-income people view the potential environmental consequences of human interventions as extremely dangerous, more so than high-income people (Lo, 2014).

## Materials and methods

### Historical context

Between 2011 and 2019, Louvain Coopération has developed as a part of its Food and Economic Security program a specific approach to systematically take into account the environment in its interventions, translated into two Environmental Integration Tools (EITs): the EIT-Program and the EIT-Producer, registered under a Creative Commons License (CC BY-SA: Attribution-ShareAlike). From a single variant tested in 2012 (EIT Approach version 1) through EIT Approach version 2/2015, EIT Approach version 3/2017, EIT Approach version 4/2018 and on to the final version validated in 2019 (EIT Approach version 5), an intense work of progressive improvements has been implemented after a process of field experiments (in African countries – Benin, Burundi, Madagascar, Congo and Togo -, in Andean America – Bolivia and Peru – and in Asia – Cambodia – where Louvain Coopération operates) and internal and external exchanges, with multiple actors, especially those of the Belgian development cooperation (Henin and Uglione, 2021). The EIT Approach has been the subject of an intense dissemination campaign in the Belgian development cooperation but also in academia. By the time the mid-term evaluations of the Food and Economic Security program 2017–2021 were finalized, the IET Approach had been disseminated in all ongoing Food and Economic Security program over the 2017–2020 period and in other institutions. This large-scale diffusion has been fundamental for the continuous improvement of the EIT Approach, but also for the formation of staff who, in addition to mastering its approach and its tools, have appropriated them in a creative way (Henin and Uglione, 2022).

### Document analysis

Document Analysis is a method of data collection used in different fields of research as a supplement to other methods or as a primary method (Coustaty et al., 2011; Metag et al., 2016; Cardno, 2018; Price et al., 2021 and others). The material used in a Document Analysis can be in printed or electronic format (Fischer, 2006). This provides a very wide applicability to this method, and this is one of its advantages (Bowen, 2009). The great diversity of documents generally available to a researcher should be used to benefit the credibility of his or her research. This diversity is especially important in expanding the contextual variables of the data (Price et al., 2021). Documents are stable and “non-reactive” data sources (Bowen, 2009). They can be reviewed repeatedly without being transformed by the research process itself. However, this stability requires a lot of work in compiling and interpreting the information, which sometimes depends on a much broader investigation than initially expected, with the inclusion of additional documents (Bowen, 2009). Document Analysis requires revised planning throughout the research process, as having an evolving list of documents to explore; paying continuous attention to the peculiarities and possible language or cultural barriers of the documents, being current on the topics of the documents and taking into account ethical issues (such as document confidentiality; O’Leary, 2014).

### Process

*In what socio-historical context was the EIT Approach conceived? Who were the main actors in this process? What ideas and concepts have guided its different versions? How was it disseminated internally and externally? What are the impacts of this innovation on Louvain Coopération’s programs and areas of intervention?* Based on these initial research questions, five categories were established to guide the selection of documents:

- Materials concerning the construction process of the EIT Approach;
- Materials concerning the diffusion and appropriation process and impact of the EIT Approach;
- Versions and variants of the EIT Approach;
- Food and Economic Security/FES program reports and
- Others.

A total of 46 documents from the virtual library of Louvain Coopération were analyzed. The selection of documents was based on the principle of saturation (Saunders et al., 2018). A list of these documents is presented in Table 1.

**Table 1:** List of documents.

Category	Title
A	01 Synthèse de Mémoire 02 Stratégie de Visibilité et Reconnaissance 03 Stage Salima Kempenaer en Bolivie 04 Manuel d’Intégration Environnementale/Union Européenne version 2006 05 Manuel d’Intégration Environnementale/Union Européenne version 2009 06 Outil Klimos 07 Mission de l’Assistante Junior Delphine Latinis en Amérique Andine 08 Rapport de Mission au Bénin 09 Communication Interne Louvain Coopération
B	10 Formation donnée lors du Stage Méthodologique en Conception de Projets pour le Développement Durable/Sénégal, RDC, Bénin, Burkina et Haïti 11 Présentation/Agricongo, 2017 12 Formation/l’ONG syndicale MSI, 2018 13 Présentation (Solidarité Internationale pour le Développement et l’Investissement-SIDI/France et Société Wallonne de Financement-Sowalfin), 2019 14 Présentation lors de la mission de Vincent Henin à Madagascar, 2018 15 Présentation à l’Université de Parakou, 2019 16 Rapport Formation Burundi 17 Méthodologie Formation Burundi 18 Intégration de la Préoccupation Environnementale en Coopération au Développement/Présentation 19 Schéma Programme DGD 2022–2026 20 Synthèse Réunion Louvain Coopération avec l’Association Belge APEFE
C	21 Approche Outil d’intégration Environnemental – Présentation 22 Outil d’Intégration Environnementale version 1 23 Outil d’Intégration Environnementale version 2 24 Méthodologie de l’Outil d’Intégration de l’Environnement dans le Programme SAE de Louvain Coopération 25 Outil d’Intégration Environnementale au Niveau Institutionnel version 3 26 Outil d’Intégration Environnementale au Niveau des Bénéficiaires version 3 27 Méthodologie de l’Outil d’Intégration Environnementale au Niveau Institutionnel version 3 28 Méthodologie de l’Outil d’Intégration Environnementale au Niveau des Bénéficiaires version 3 29 Outil d’Intégration Environnementale Programme/OIE-Programme – version 4 30 Outil d’Intégration Environnementale Producteur/OIE-Producteur version 4 31 Approche Outil d’Intégration Environnemental/OIE Programme version 5 32 Approche Outil d’Intégration Environnemental/OIE Producteur/Productrice version 5
D	33 Rapport Narratif AMSANA 34 Rapport d’Évaluation à Mi-parcours SAE Uni4Coop Bénin 35 Rapport d’Évaluation à Mi-parcours SAE Uni4Coop Bolivie 36 Rapport d’Évaluation à Mi-parcours SAE Uni4Coop République Démocratique du Congo 37 Rapport Interne SAE République Démocratique du Congo 38 Rapport Interne SAE Madagascar 39 Rapport Interne SAE Bénin 40 Rapport Interne SAE Bolivie 41 Rapport Interne SAE Togo
E	42 Synthèse du Chantier 43 Synthèse Réunion Version 3 44 Liste OIE Remplis au Sein de Programmes SAE 45 Rapport Mensuel du Programme Petit Entreprenariat Rural 2 (fourni par ULB-Coopération à Louvain Coopération) 46 Synthèse Mission Outils d’Intégration Environnementale-OIE au Siège

Initially, this material was read superficially in order to gain a better understanding and sensitivity to the vocabulary and context in which the documents were produced (Altheide, 2000). This was followed by a process of immersion, reading and re-reading of the documents until familiarity with the data was achieved (Bowen, 2009). This immersion allowed the classification of the documents according to their most relevant themes. The guiding/initial questions of the research were always kept as a reference (Lincoln and Guba, 1985). From this classification emerged three main analytical axis:

- Axis 1 – The construction of the EIT Approach.
- Axis 2 – The diffusion and appropriation of the EIT Approach.
- Axis 3 – The impact of the EIT Approach.

The documents were read several times, as part of an inductive work to identify relevant topics based on the information contained in those documents, as well as on the practical and theoretical knowledge of the researchers (Jodelet, 2003). This led to the emerging of leads that guided the definition of categories which guided the analysis within each axis in a more straightforward manner. Within each axis, a lexical approach to Textual Data Analysis guided the grouping of information based on what it says (Moscarola, 2018). This grouped information was described, and finally, a synthesis of each axis was constructed from inferences of this information.

## Results

### Conceptual and methodological aspects of EIT producer

The results concerning the conceptual and methodological aspects of the EIT Producer were found mainly in the analyses in Axis 1. The most important documentary sources for this analysis were:

1. Materials concerning the construction process of the EIT Approach;
2. Versions and variants of the EIT Approach and
3. Others.

The construction of the EIT Approach was a large and gradual process, from a single tool to a version with two tool variants, the EIT Program and the EIT Producer. Successive changes in form, content and methodological aspects were made throughout the process of developing the approach. Multi-stakeholder participation and field experimentation were the basis for this process. Why create a new methodology for environmental integration? Louvain Coopération’s ambition was to develop an approach that would play an important role in the conception of programs/projects (concepts, objectives, etc.), rather than being a tool focused on their evaluation. A practical methodology, capable of being a source of information and education. In addition, this new approach should drive a systematic environmental integration, present in all phases of a project/program. The first version of the EIT Approach was launched in 2012 as a single questionnaire where the questions are articulated around two main pillars:

1. Influences of the environment on projects and
2. Influences of project’s actions on the environment.

The content analysis of these questions shows ideas regarding the intrinsic link between environmental, social, and economic factors (poverty and others), forces underlying environmental pressures (legal issues, job opportunities, and others) and groups particularly vulnerable to environmental issues. “*Was the environment properly taken into account in the initial diagnosis of the problems to be solved?*” (question number 1 from Outil d’Intégration Environnementale version 1, page 1, doc. 22 – List of Document). In this question, the methodological strategy is not only to ask whether the environment is taken into account in the diagnosis phase. The goal is to stimulate reflection on the importance of taking it into account. This is a methodological perspective in which the data collection on the environmental actions/behaviors of the actors is combined with the stimulus to the reflexivity of these actors concerning these actions/behaviors.

“*The tool* [Environmental Integration Tool, now EIT Approach] *can be compared to a common thread in order to improve the reflections and decision making at every phase of the Project Cycle*” (Méthodologie de l’Outil d’Intégration de l’Environnement dans le Programme SAE de Louvain Coopération, page 1, doc. 24 – List of Document).

This and other explanations emphasize the transversality of the integration envisaged by the approach and the methodological dynamic through which it must be achieved, i.e., provoking the reflection that will lead to a decision.

The 2015 version has been tested extensively through the FES program. This process resulted in the third version, launched in 2017, with two variants: EIT Institutional and EIT Beneficiary. This innovation reflects the difference between a tool designed for those who manage projects and a tool developed for those whom these projects are intended to reach, actors in local economy (individuals, families, associative groups, etc.), participants of the FES program.

This version presents substantial changes. In both variants, concepts of sustainable development and environmental protection are added, as well as a more detailed contextualization of the expectations of the tools. The semi-structured questionnaire remains the basis for both variants, but the structure (the steps and the content of the questions) and the methodology are different in each one. The EIT Institutional reproduces the dynamics of the previous version. This application covers all phases of the Project Cycle Management (identification, formulation, implementation and evaluation). In the EIT Beneficiary, the application takes place only in the implementation phase and is intended to be accomplished in four stages: Environmental Diagnosis, Diagnostic Analysis, Self-Determined Commitment, and Implementation of Commitment.

“*They* [EIT Institutional and EIT Beneficiary] *offer us two different points of view, the EIT Beneficiary arises from a shortcoming of the EIT-Institutional, which only takes into account the vision of the technicians of the project and not the vision of the beneficiaries*” (Synthèse réunion version 3, np, doc. 43 – List de Document).

“*The 3rd stage concerns the commitments from the beneficiary. A second interview is proposed to him to discuss the results of the analysis* [Diagnostic Analysis] *and to and to establish the pro-environment commitments*” (Méthodologie de l’Outil d’Intégration Environnementale au Niveau des Bénéficiaires, page 3, doc. 28 – List of Document).

The self-determined commitment is therefore integrated into this third version, as a step after the analysis work, and carried out through an interview between the team managing the project and the beneficiary (an agent of the local economy).

In the 4th version, of 2018, the EIT Beneficiary becomes EIT Producer. The collective and dialogical dynamic behind its methodology is highlighted in this version.

“*This tool* [EIT Producer] *is designed to structure a dialogue, a reflection at the environmental level between an economic actor responsible for a productive activity supported by one of our projects/programs, and the technical team of the project/program*” (Outil d’Intégration Environnementale/OIE-Producteur version 4, page 2, doc. 30 – List of Document).

In 2019, a new version [current EIT Approach, with its two variants EIT Program and EIT Producer] is presented and the whole construction process reaches its stabilization. The steps of the EIT Producer become: Environmental Diagnosis (step 1), Commitments (of the economic agent, step 2) and Commitments Implementation (step 3). The Environmental Diagnosis is carried out through a semi-structured questionnaire, understood as a “reflection guide” used during a meeting between the technical team and the producer, or by the producer alone, in an exercise of self-reflection.

#### Self-determined commitments and Pro-environmental behavior

“(…) *highlighting mutual effects between economic/productive activity and environment, the willingness of the economic agent to better take environmental issues into account, and his/her capacities and needs to commit to*” (Approche Outil d’intégration Environnemental – Présentation, page 3, doc. 21 – List of Document).

“*The idea is to highlight the producer’s knowledge and perceptions of the environment, as well as the main mutual relationships (positive or negative) between his activity and the environment*.” (Approche Outil d’Intégration Environnemental – OIE Producteur/Productrice version 5, page 3, doc. 32 – List of Document).

The topics/parts of the environmental diagnosis are:

- The terms of use: identification of the producer, description of possible third parties taking part in the diagnosis and their role (as observers, facilitators, etc.), and determination of who assigns the indicator scores;
- A brief description of the producer’s activity;
- A brief description of the environmental context, which provides a baseline of the environmental situation in the activity or area;
- A brief description of the mutual effects between the environment and the producer’s activity;
- A semi-structured questionnaire for themes:

1. The effects of the environment on the producer’s activity;
2. The effects of the producer’s activity on the environment;
3. The producer’s willingness to commit for the environment and
4. The producer’s capacities and needs to commit;

- A summary of all scores, as well as the main actions identified to improve the environmental situation of the activity and
- The lessons learned during this process, highlighting what the key actors gained from the diagnosis, and potential proposals for improvement.

**Figure 1:**
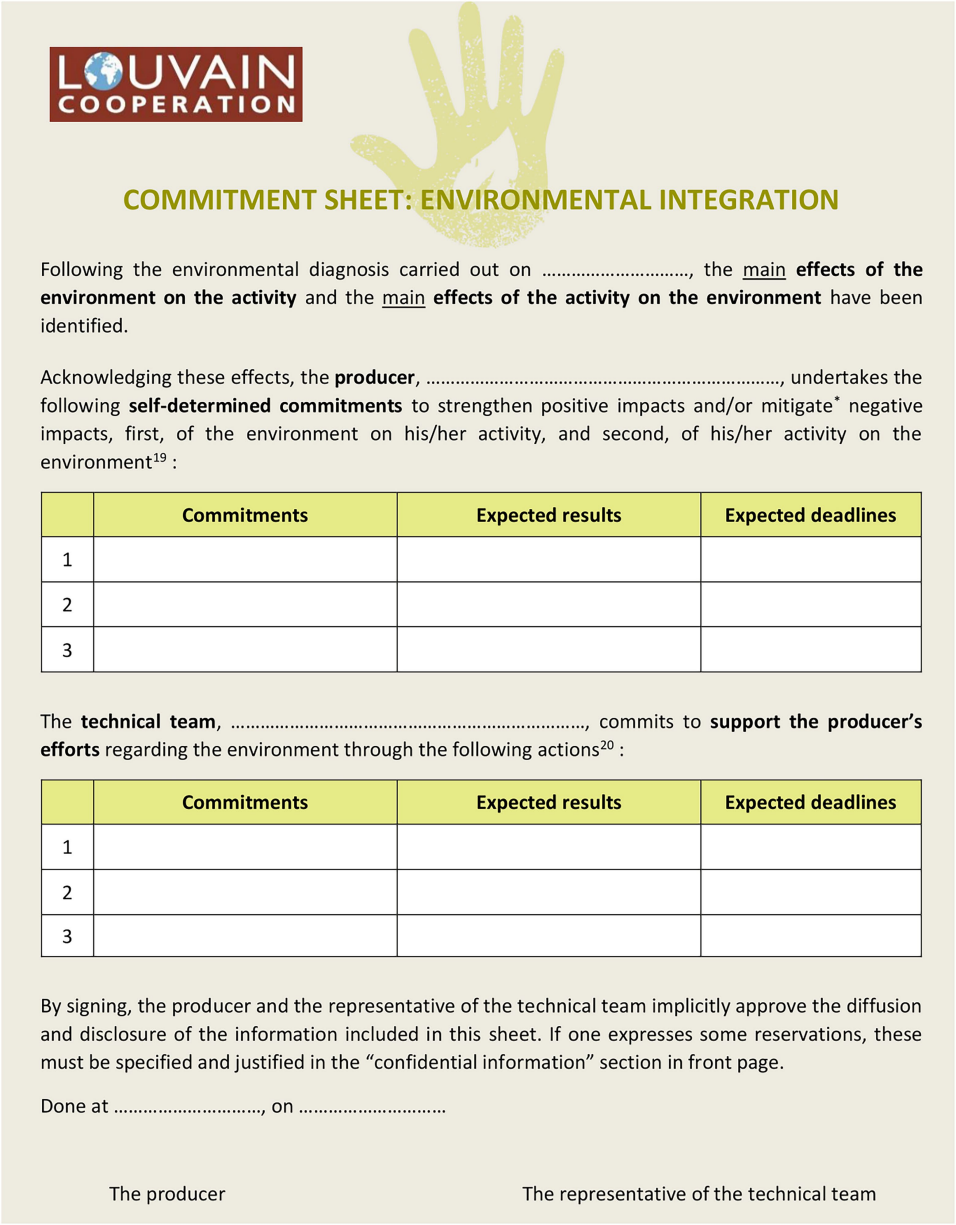
The commitment sheet/EIT producer.

After the diagnosis, the local economic actor is invited to engage himself to implement actions in order to strengthen the positive impacts and/or mitigate the negative impacts of the environment on his activity, and of his activity on the environment. This is the step 2 of the EIT Producer, the one of the Commitments. The technical team proposes a sheet which the producer chooses to use or not (see Figure 1).

The commitments can be made verbally as well as in writing, individually or collectively.

“*It is essential that the producer personally determines his commitments, without being constrained by the technical team. The technical team is responsible for determining the modalities of support and follow-up with the producer. The deadlines set are determined according to the producer’s possibilities and the project/program schedule*” (Approche Outil d’Intégration Environnemental-OIE Producteur/Productrice – version 5, page 18, doc. 32 – List of Document).

The third and final step of the EIT Producer is the Commitments Implementation. The actions resulting from the commitments are evaluated according to the deadlines determined during the commitment step. A new cycle of diagnosis and eventual new commitments may take place after some time, depending on the progress of the implementation of the actions.

### Implementation process and impact of the EIT producer

The analyses on Axis 2 and Axis 3 were the basis for the results found about implementation process and impact of the EIT Producer. The material analyzed was extracted from:

1. Documents concerning the diffusion and appropriation process and impact of the EIT Approach,
2. Reports from the Food and Economic Security program and
3. Others.

In 2020, the EIT Approach had been disseminated across all of its ongoing projects up to that point. The EIT Approach was presented to all FES technical teams in the different countries where projects are implemented. Training sessions were organized, implemented using participatory methodologies and didactic materials with multiple information and practical examples. These teams actively participated in the improvement and adaptation of the EIT Approach according to the needs and circumstances inherent to each project and context where they were being implemented. By 2020, the EIT Approach was already part of the common vocabulary of all FES program projects. It was mentioned in all FES program Internal Reports in 2019 and in all Interim Evaluation Reports of Uni4Coop (the NGO Consortium of Francophone Belgian universities through which Louvain Cooperation is subsidized by DGD) projects carried out between 2018 and 2020. In most cases, it is presented as an intervention strategy, linked to the most important objectives and actions of the project and defined mainly as:

- A method of study/data collection/interview that allows the environment/environmental issues to be taken into account in the project and
- An approach that allows for self-determined commitments and actions by local economic actors regarding the environment.

Other definitions, less prominent in the reports, are:

- Training and exchange method between local technical teams and
- Awareness and empowerment of actors (partners, local economic actors) for environmental protection/climate change

**Figure 2:**
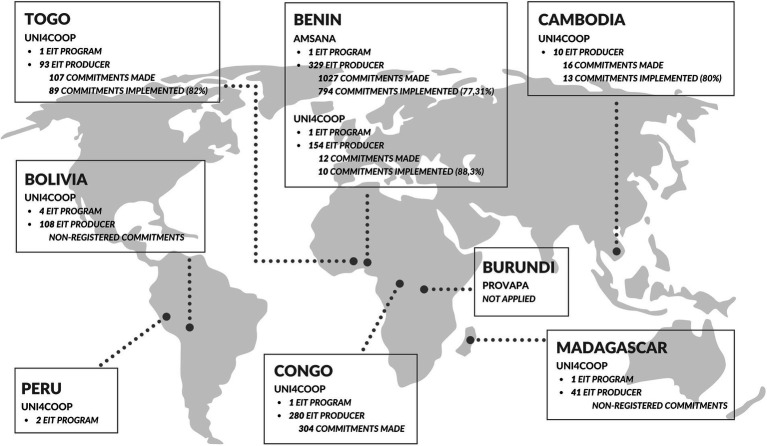
Implementation of the EIT approach in the FES program between 2016 and 2020.

The implementation of EIT Approach took place through different actions, rhythms and intensities in each project, according to their particularities and constraints. The application of its tools (EIT Program et EIT Producer) varied significantly within the projects. Figure 2 shows the geographical regions in which the EIT Approach was implemented within the FES program, the number of commitments made and the number of commitments implemented.

The EIT approach was not applied in all projects, but in those where it was, the number of EIT Producer applications was significantly higher than those of the EIT Program. The only exception was in Peru (where there were two EIT Programs and no EIT Producer). In this country there has been no implementation of the FES program since 2017, when the EIT Beneficial (now EIT Producer) was developed.

Under the Food and Economic Security Program/FES, the application of the 1015 EIT Producer resulted in 1049 self-determined commitments by the local producers. From this, 906 resulted on pro-environmental actions. Some examples of these actions can be seen in initiatives in Benin, such as crop rotation, composting and usage of organic inputs and pesticides, integrated management of crop pests and diseases, reforestation, the use of living hedges and many others. In this country, this application generated 794 pro-environmental actions, which represents 77.31% of the commitments. The application of the EIT-Producer in Togo generated 89 pro-environmental actions by economic agents, which corresponds to 82% of the total self-determined commitments undertaken by these actors.

The number of EIT Producer applications in each country varied significantly, as did the impact in terms of commitments (see Figure 2). In some, commitments were made, and this resulted in actions that were followed up by the technical teams. In others, the commitments steps were not register and/or followed up, which makes it impossible to know whether or not the commitments were made. These and other variations have characterized the implementation of the EIT Producerand its impacts, related to different reasons and particularities of each project. An example is illustrated in the excerpt from narrative report FES program AMSANA Benin (doc. 33 – List of Document):

“*The decrease in the rate of implemented commitments compared to the previous year is due to the 400 new commitments made by the producers of the 3rd call* [call for participation in the FES program] *whose implementation will be effective next June* [a few months after the evaluation regarding this report]” (page 42,).

Evaluations of projects where the EIT Producer has been implemented emphasize the scale of its impacts.

“(…) *It is important to note that the EIT, besides being an awareness tool, it is also an instrument for accountability and appropriation of mechanisms for an efficient and sustainable environmental management.*” (internal project report FES program Uni4Coop Congo, p. 38, doc. 37 – List of Document).

“*The implementation of the EIT Approach allows for diagnostic, commitment, monitoring, and evaluation actions in a few steps*” (mi-term evaluation report FES program Uni4Coop Bolivie, p. 64, doc. 35 – List of Document).

Regarding the difficulties and obstacles in the implementation of the EIT Producer, the excerpt from internal report FES program Uni4Coop Madagascar (doc. 38 – List of Document) exemplifies reasons attributed by the technical teams:

“(…) *lack of time and personnel* (…) *difficulty in translating the concepts and terms of the tools into the official national language and local dialect*” (pages 14 and 53).

## Discussion

The qualitative research on the construction, dissemination, appropriation and impact of the EIT Approach has made it possible to analyze an important and rich documentation. These documents testify to the trajectory and maturing of the rationalities that guide the participation of the Belgian NGO Louvain Coopération in international development cooperation. This is a plural material, with different structures, profile and objectives. They have been written by different people and groups, stakeholders of different hierarchical levels of the programs and projects of Louvain Coopération. They contain very pronounced nuances of language. This was crucial to the diversity of data and complexity of the analyses, both fundamental to the credibility of the research. All documents of the virtual library of Louvain Coopération concerning the EIT Approach were made available for the research, which greatly facilitated the research process. All documents that contained significant data on the research questions were selected, but overall, the data from the 46 selected documents did not saturate the questions. They provided highly relevant leads to answers to this research questions, and they also provided leads on the need for further research.

From the historical point of view of the EIT Producer (Axis 1 – building process), the commitments have been the element through which a conceptual, epistemological and political shift has occurred within Louvain Coopération initiatives regarding environmental integration. This led to a change of course, with a view to adopting a coherent approach and working in complementarity with other actors. This perspective is at the origin and basis of the current international development cooperation, endorsed and ratified in its most notorious documents. Louvain Coopération’s impetus for building an environmental integration approach was to extend its functionality in the context of the objectives of its development cooperation programs. This integration should promote information and education. For whom? To the actors to whom its projects are mainly directed, local producers in peripheral and rural areas of the countries of the South. Why? To positively influence the awareness of its actors about the reciprocal effects between their production activities and the environment. And how to do this surmounting the risk that this integration is not exogenous, without meaning or appeal to these actors, pointed out by evaluators of cooperation programs in development? The main innovation of the EIT Approach version 2017, EIT Beneficial, brings an answer, that is, taking into account, seriously and concretely, the local actors. In this version, self-determined commitment is integrated into the EIT Approach Beneficial methodology.

That raising awareness of environmental protection is a very difficult task because it implies a change of mentalities and behaviors that are hegemonic and stimulated in the global consumer society, but also because in general it implies a personal engagement with a world that is not the one in which we live, but which is part of the future (Kempf, 2007; Moser, 2009; Bonnefoy et al., 2010; Girandola and Joule, 2012). The analysis of the documents of Axis 1 shows that the insertion of self-determined commitments has been the main strategy to achieve an approach of environmental integration capable of having a significant influence on the awareness of the actors and the whole psychological process that it implies. The EIT Producer is focused on the commitments of local producers. Self-determined commitments are clearly the main methodological dispositive of this variant. The proposed diagnosis (step 1 of the EIT Producer) aims at mobilizing the actors’ intention to act. The topics/parts of the questionnaire used in the Environmental Diagnosis lets us presume its objective of promoting a rather broad and provocative reflection, including the survey of information about the producer’s activities, the environmental context of these activities, the mutual effects between the environment and the producer’s activity. The producer’s willingness to engage with the environment, as well as his abilities and needs to do so, are also topics addressed in this step. In its final part, the focus is on the lessons learned from the diagnostics process. A set of topicals/parts that indicates that this step (Environmental Diagnosis) is conceived as a path leading to further, concerning these commitments, rather than as a moment with ends in itself.

People are not passive products of the circumstances of their lives (Bandura, 2006) and are able to determine and even change circumstances, within social and interpersonal contexts that are more or less supportive of this. The dialogical aspect that can be seen since the first versions of the EIT Approach is clearly reinforced in the EIT Producer application methodology. The interview procedure is, above all, a mark of the importance of an interpersonal environment in which the team provides information and encourages a discussion that favors the producers’ intention to act. The behavior change implies an intention to act that depends on convincing arguments about the benefits of change. Studies in various fields of application converge on this point (Nguyen, 2015; Saari et al., 2021 and others). The dialogical, collective interview is the main methodological tool of the EIT Producer. This interview frames an interpersonal relationship, in which the team is responsible for supporting the producers in their cognitive process to eliminate or significantly reduce their doubts and ambivalence about the change and the new behavior to adopt (Prochaska and DiClemente, 1992). Individuals make reasoned decisions (Ajzen, 1991). The methodological dynamics of applying steps 1 and 2 of the EIT Producer acts on the producers’ decision-making process. It acts on the cognitive representation of these actors’ willingness to adopt pro-environmental behavior (Ajzen, 1991) and favors their intention to act in favor of the environment by promoting social and interpersonal conditions that facilitate in them the psychological mechanism of predicting the consequences of these actions before deciding to take them and how easy or difficult it is to carry them out.

The application of the 1015 EIT Producer resulted in 1049 self-determined commitments by the local producers, made of intentions to act (e.g., plant trees around river areas, eliminate the use of plastics in the packaging process, reduce the use of chemical pesticide and many others) eventually listed and signed by the producers (registered in the commitments sheet form). The intention to act is the direct antecedent of behavior and the stronger the intention, the more effort an individual will make to adopt pro-environmental behavior (Ajzen, 1991; Steg and Nordlund, 2013). The procedure of listing the desired actions and taking the commitment to implement them can be considered as a methodological tool of the EIT Producer that operates in reinforcing the producers’ intention to act and thus increases the likelihood that they will actually implement them. Intention is an important predictor of people’s ecological behavior (Steg and Nordlund, 2013). Self-commitment prompts the EIT Producer to positively impact the adoption of pro-environmental behaviors and the 906 implemented actions (from the total of 1046 commitments) corroborate that. The non-mandatory use of the commitment form is a norm with the EIT Approach, in line with the principle of “openness” and the autonomy of the actors, which is the basis of the approach, defended in all of its versions. This aspect, however, does not favor the quantitative analysis of its impacts. A multi-method qualitative perspective on data collection combined with quantitative methods would be very beneficial for further research.

## Conclusion

The Document Analysis on EIT Approach shows that self-determined commitment is the nodal strategy of the EIT Producer, and that it primarily aims to affect the intention of local producers to act. The results indicate that the self-determined commitments undertaken by local economic agents is a factor that promotes the adoption of pro-environmental behavior. This is in line with theories and studies that assume intention as an internal variable that influences people’s environmental behavior patterns (Ajzen, 1991, 2011; Prochaska and DiClemente, 1992; Farrow et al., 2017; Iman et al., 2019; Schill et al., 2019). The EIT Producer encourages self-determined commitments by local economic agents, representing a crucial methodological aspect for generating pro-environmental behavior.

North–South development cooperation is a complex institution and involves an immense multiplicity of actors. Its foundations and priorities are not at all convergent, and the sectors in which it intervenes are increasingly dispersed, affecting very different scales (Étienne, 2007). Its challenges are enormous and multidimensional, but in all sectors and scales it is necessary to change both the ideas and the practices of the actors (European Commission, 2011). The EIT Approach, especially through its EIT Producer variant, constitutes a relevant methodology for projects committed to sustainable development.

## Data availability statement

The raw data supporting the conclusions of this article will be made available by the authors, without undue reservation.

## Author contributions

VH and PU contributed to the conception and design of the study, organized the database, and wrote sections of the manuscript, contributed to the manuscript revision, read, and approved the submitted version. All authors contributed to the article and approved the submitted version.

## Conflict of interest

The authors declare that the research was conducted in the absence of any commercial or financial relationships that could be construed as a potential conflict of interest.

## Publisher’s note

All claims expressed in this article are solely those of the authors and do not necessarily represent those of their affiliated organizations, or those of the publisher, the editors and the reviewers. Any product that may be evaluated in this article, or claim that may be made by its manufacturer, is not guaranteed or endorsed by the publisher.
